# Supplementary material for: Comparative genomics provides new insights into the diversity, physiology, and sexuality of the only industrially exploited tremellomycete: Phaffia rhodozyma
Source: BMC Genomics. 2016 Nov 9;17:901. doi: 10.1186/s12864-016-3244-7 (PMC5103461; doi:10.1186/s12864-016-3244-7)
Supplement: Additional file 6: — List of orphan genes with links to PFAM (related to Additional file 1: Table S1). (ZIP 1428 kb) [file 12864_2016_3244_MOESM6_ESM.zip › BLAST_HTML_FTR/G01966_P.html]

BLAST Search Results


```
BLASTP 2.2.27+


Reference:
Stephen F. Altschul, Thomas L. Madden, Alejandro A. Schäffer,
Jinghui Zhang, Zheng Zhang, Webb Miller, and David J. Lipman (1997),
"Gapped BLAST and PSI-BLAST: a new generation of protein database
search programs", Nucleic Acids Res. 25:3389-3402.


Reference for
composition-based statistics:
Alejandro A. Schäffer, L. Aravind, Thomas L. Madden, Sergei
Shavirin, John L. Spouge, Yuri I. Wolf, Eugene V. Koonin, and
Stephen F. Altschul (2001), "Improving the accuracy of PSI-BLAST
protein database searches with composition-based statistics and
other refinements", Nucleic Acids Res. 29:2994-3005.


Database: nr
           71,551,133 sequences; 26,053,659,533 total letters


Query= G01966_P

Length=447
                                                                      Score     E
Sequences producing significant alignments:                          (Bits)  Value

emb|CDZ96808.1|  hypothetical protein [Xanthophyllomyces dendrorh...   791    0.0  


 >emb|CDZ96808.1| hypothetical protein [Xanthophyllomyces dendrorhous]
Length=463

 Score =  791 bits (2044),  Expect = 0.0, Method: Compositional matrix adjust.
 Identities = 425/463 (92%), Positives = 426/463 (92%), Gaps = 31/463 (7%)

Query  15   MTVHPSSSTSS-------ATIGLV------------------------DRAQVPVSIDSS  43
            M +HP  S          ATIGLV                        DRAQVPVSIDSS
Sbjct  1    MYIHPRQSDGLFSSSTSSATIGLVVCLVLIGVSAVIGVSLLYYRCRRKDRAQVPVSIDSS  60

Query  44   MPTMKITFLSPPSSTSSPIRMELVPASTGHSHETDCQAAHSSHSRIGLVEAGGGGGGACC  103
            MPTMKITFLSPPSSTSSPIRMELVPASTGHSHETDCQAAHSSHSRIGLVEAGGGGGGACC
Sbjct  61   MPTMKITFLSPPSSTSSPIRMELVPASTGHSHETDCQAAHSSHSRIGLVEAGGGGGGACC  120

Query  104  GVSAEDHRTRSILSDDVSLEEGVGDWSQVQSYEILSPTLSPDNHRFSSFEFNSQKGMDVP  163
            GVSAEDHRTRSILSDDVSLEEGVGDWSQVQSYEILSPTLSPDNHRFSSFEFNSQKGMDVP
Sbjct  121  GVSAEDHRTRSILSDDVSLEEGVGDWSQVQSYEILSPTLSPDNHRFSSFEFNSQKGMDVP  180

Query  164  SALGLETRPNLSRTMSLSSDSFYAHYPSRQLEDSAHQVHRHTVQPGFSRSVSFGAYGEPT  223
            SALGLETRPNLSRTMSLSSDSFYAHYPSRQLEDSAHQVHRHTVQPGFSRSVSFGAYGEPT
Sbjct  181  SALGLETRPNLSRTMSLSSDSFYAHYPSRQLEDSAHQVHRHTVQPGFSRSVSFGAYGEPT  240

Query  224  ARPRPTPNHSYSYSHQSYGTPSFYRFTSAPPQTSPVSSEDSPSDFLPASPSISYHRPTIS  283
            ARPRPTPNHSYSYSHQSYGTPSFYRFTSAPPQTSPVSSEDSPSDFLPASPSISYHRPTIS
Sbjct  241  ARPRPTPNHSYSYSHQSYGTPSFYRFTSAPPQTSPVSSEDSPSDFLPASPSISYHRPTIS  300

Query  284  KHRRSISMPDALPFTPSSSPPMTTASHFAWSSSSPIFPLPPSLHCANSNTIPALMRQTKA  343
            KHRRSISMPDALPFTPSSSPPMTTASHFAWSSSSPIFPLPPSLHCANSNTIPALMRQTKA
Sbjct  301  KHRRSISMPDALPFTPSSSPPMTTASHFAWSSSSPIFPLPPSLHCANSNTIPALMRQTKA  360

Query  344  MEFESASSRVFTDGYFSDLYERPIEHLSGNSLLGESLIKSTRSSVATTHLDQEAEPVWDH  403
            MEFESASSRVFTDGYFSDLYERPIEHLSGNSLLGESLIKSTRSSVATTHLDQEAEPVWDH
Sbjct  361  MEFESASSRVFTDGYFSDLYERPIEHLSGNSLLGESLIKSTRSSVATTHLDQEAEPVWDH  420

Query  404  GRAGFSRNFSQEREEDRRYHEGEERLPPGYEQSEESFNGSTMV  446
            GRAGFSRNFSQEREEDRRYHEGEERLPPGYEQSEESFNGSTMV
Sbjct  421  GRAGFSRNFSQEREEDRRYHEGEERLPPGYEQSEESFNGSTMV  463


Lambda      K        H        a         alpha
   0.313    0.126    0.374    0.792     4.96 

Gapped
Lambda      K        H        a         alpha    sigma
   0.267   0.0410    0.140     1.90     42.6     43.6 

Effective search space used: 4405192019943


  Database: nr
    Posted date:  Sep 23, 2015 12:05 AM
  Number of letters in database: 26,053,659,533
  Number of sequences in database:  71,551,133


Matrix: BLOSUM62
Gap Penalties: Existence: 11, Extension: 1
Neighboring words threshold: 11
Window for multiple hits: 40
```
